# Food insecurity and self-rated health in rural Nicaraguan women of reproductive age: a cross-sectional study

**DOI:** 10.1186/s12939-018-0854-5

**Published:** 2018-09-18

**Authors:** Wilton Pérez, Mariela Contreras, Rodolfo Peña, Elmer Zelaya, Lars-Åke Persson, Carina Källestål

**Affiliations:** 1 International Maternal and Child Health (IMCH), Department of Women’s and Children’s Health, Uppsala University, Uppsala, Sweden; 2 Pan American Health Organization, Tegucigalpa, Honduras; 3 Asociación para el Desarrollo Económico y Social de El Espino (APRODESE), Cinco Pinos, Nicaragua; 4 Department of Disease Control, London School of Hygiene & Tropical Medicine, London, UK

**Keywords:** Self-rated health, Food insecurity, Capability approach, Nicaragua

## Abstract

**Background:**

Access to food is a basic necessity, and food insecurity may impair the individual’s well-being and health. Self-rated health measurements have frequently been used to assess population health. Little is known, however, as to whether food security is associated with self-rated health in low- and middle-income settings. This study aims at analyzing the association between food security and self-rated health among non-pregnant women of reproductive age in a rural Nicaraguan setting.

**Methods:**

Data was taken from the 2014 update of a health and demographic surveillance system in the municipalities of *Los Cuatro Santos* in northwestern Nicaragua. Fieldworkers interviewed women about their self-rated health using a 5-point Likert scale. Food insecurity was assessed by the household food insecurity access (HFIAS) scale. A multilevel Poisson random-intercept model was used to calculate the prevalence ratio.

**Results:**

The survey included 5866 women. In total, 89% were food insecure, and 48% had poor self-rated health. Food insecurity was associated with poor self-rated health, and remained so after adjustment for potential confounders and accounting for community dependency.

**Conclusion:**

In this Nicaraguan resource-limited setting, there was an association between food insecurity and poor self-rated health. Food insecurity is a facet of poverty and measures an important missing capability directly related to health.

## Background

Hunger and undernutrition were reduced during the Millennium Development Goal era of 1990–2015. However, food insecurity increased from 2013 to 2016, affecting 815 million people with 689 million people severely food insecure. During that three-year period, food insecurity increased from 4.7 to 6.4% (42 million) in Latin America [1].

The second Sustainable Development Goal (SDG) aims to end hunger, achieve food security, improve nutrition, and promote sustainable agriculture by 2030. It includes targets to end all forms of malnutrition during childhood, adolescence, pregnancy, and lactation [2]. Malnutrition is shown both as undernourishment and overweight or obesity, which is further associated with non-communicable diseases [3]. These diseases generally appear through “socially transmitted conditions”, indicating the influence of social and food environmental factors on dietary behavior changes linked to the nutrition transition [3, 4]. Therefore, food security is a necessary component to achieve the second SDG and ensure women’s health (SDG 3).

The Food and Agricultural Organization defines food security as “a situation that exists when all people, at all times, have physical, social and economic access to sufficient, safe and nutritious food that meets their dietary needs and food preferences for an active and healthy life” [5]. There are two categories of indicators of food insecurity. The first is based on the adequacy of food consumption, and the second is based on the severity of constrained food access. The latter scales were based on ethnographic research and have been shown to have good feasibility and generate valid and comparable results across countries [6–8]. These scales measure food insecurity as part of the multidimensionality of poverty. These characteristics are important as food insecurity might affect well-being beyond the negative influences on nutritional status [8].

Studies in the United States have shown an association between food insecurity and reduced well-being, mental health problems, depression, hypertension, hyperlipidemia, and sleep deprivation [9]. The mechanisms may be direct or indirect. For instance, food insecurity was indirectly associated with obesity in American and Iranian women [10–12] and with poor mental health in low-income settings [13].

Self-rated health (SRH) is a proxy measure of health and well-being constructed from individual answers to the question “In general, would you say that your health is excellent, very good, fair, or poor?” Reporting poor to fair health is associated with increased risk of future mortality. This association has also been documented in low- and middle-income countries [14], although it has been questioned in some settings [15]. The achievement of a good SRH depends on different social determinants and the ability of individuals to get access to education, employment, food, and health care [16, 17].

### Self-rated health and the capability approach

Health and well-being is not comprehensively achieved under an economic welfare framework. Single macroeconomic monetary poverty measures used by the World Bank, such as Gross Domestic Product per capita, which classifies nations into high-, middle- and low-income countries, or the proportion of people living below the poverty line, are useful for international comparisons. For an improved understanding of the multi-dimensionality of poverty, other measures and theories, such as the capability approach, are suggested. The capability approach to poverty, initially developed by Amartya Sen, focuses on individuals, and prioritizes the freedom of choice a person has in life to achieve desired outcomes and utilities [18]. This approach strives to answer to question “What is this person able to do and be?” [19]. The literature on the capability approach is growing in the fields of development, health, and nutrition [20, 21]. In this approach, poverty is defined as an “unfreedom, the deprivation of freedoms necessary to lead a fulfilled life” [22]. For example, the lack of opportunity for women to get foods –in quantity and quality– in rural settings limits their capability to improve their nutritional status and health [23]. Enough food is often seen as one of several basic or central capabilities that otherwise might deprive a person of her dignity and wellness. Self-rated health could be seen as a measure of well-being. Thus, an association between food security and self-rated health in a region experiencing a nutrition transition, even if poverty has been notable reduced [24], could give insights to the multifaceted construction of poverty.

The aims of this study were to measure the prevalence of food insecurity and the level of self-rated health in women of reproductive ages in four communities in northwestern Nicaragua, and to analyze whether food security as a capability at the household and community levels was associated with women’s self-rated health.

## Methods

### The Nicaraguan context

Nicaragua is classified as a lower middle-income country. Poverty dropped from 42.5 to 29.6% from 2009 to 2014, which may have contributed to achieving the food security target, halving the number of hungry people in 2015 [25, 26]. However, food insecurity experience may show variations within the country. For instance, our research group in *Los Cuatro Santos* (rural settings) reported severe food insecurity among 36% of mothers to children below three years of age in 2009 [27]. Another survey in León (urban-rural) showed that 25% of mothers had moderate to severe food insecurity and highlighted an association between food insecurity and maternal distress [28]. As the country is facing a nutrition transition, even in less-resourced settings, a cross-sectional study conducted from 2007 to 2009 in rural Nicaraguan communities showed that 22% of adults were obese and 55% were overweight or obese [29].

### Study setting

*Los Cuatro Santos* are four rural municipalities in northwestern Nicaragua. In this area, a Health and Demographic Surveillance System (HDSS) was established in 2004 with a baseline survey covering socio-economic information, population composition, births, deaths, and in- and out-migration. After the baseline, three follow-up surveys were performed in 2007, 2009 and 2014 including data on social and demographic changes. In the HDSS 2014 update there were around 25,000 inhabitants, with 25 % of the population being women of reproductive age.

During the last two decades, local development strategies were implemented, including a wide range of facilitated community based activities. It is plausible that these initiatives have improved the economic, environmental and health situation in the area [24]. For instance, poverty had been reduced from 79 to 47% from 2004 to 2014. This rural economy is based on agricultural and livestock production, but recently small service businesses have been developed. Seven percent of the economically active population had migrated to another country due to unemployment and economic difficulties [22].

### Study population and design

The 2014 follow-up survey in the HDSS included information on food security and self-rated health of all women of reproductive age (15–49 years). Women with physical or mental conditions that made interviews difficult were excluded (0.3%). We also excluded pregnant women from the present study (4%). For the purposes of this study, the design is cross-sectional.

### Household food security

The nine-item household food insecurity access scale (HFIAS), version 3, was used [7]. The respondent was the person responsible for household expenditures and food preparation. These nine questions cover experiences in the household regarding 1) anxiety in the household due to lack of food; 2) inability to eat preferred food because of lack of resources; 3) limited variety of food due to lack of resources; 4) consumption of few kinds of food because of lack of resources; 5) reduction of portion sizes of meals due to lack of food; 6) consumption of fewer meals per day because of lack of food; 7) no food to eat in the household because lack of resources; 8) going to sleep at night hungry due to lack of food, and 9) days of hunger because of insufficient amounts of food to eat. For each affirmative answer, the person provided additional information on the frequency on a four-point scale (never, rarely, sometimes, often). The HFIAS scale has been used in the study area before [27] and has been validated in various international settings [7]. The HFIAS prevalence categories 1 = Food Secure, 2 = Mildly Food Insecure Access, 3 = Moderate Food Insecure Access, and 4 = Severe Food Insecure Access were calculated and the percentage of each category analyzed using the FANTA’s procedure [7]. For modeling purposes, food security was grouped into a dichotomous variable of “food insecure” (i.e., mild, moderate and severe food insecurity) which was considered as a lack of capability, and “food secure”.

### Self-rated health

As women’s health and wellbeing are included in the 2030 SDG targets, we collected data on self-rated health in this population in the 2014 HDSS update [2]. Self-rated health was assessed with a five-point Likert scale based on the following question: “In general, how would you assess your health today?” The interviewer provided the following possible answers: very good, good, medium, bad, or very bad. The SRH question was responded to by all resident women of reproductive age, i.e., 15–49 years, at the time of interview. Responses were classified in two categories: “reported good SRH” when the response was very good, good or average; and “reported poor SRH” when the response was bad or very bad.

### Other characteristics

In each household, information was collected on education, age, and employment status of each inhabitant. The education of the women in this study was grouped into three categories: illiterate or not completed primary school, completed primary school or not completed secondary, and completed secondary school or beyond. Age was measured in years at the time of interview and categorized as less than 20 years, 20–29, 30–39, and 40 years or more. The employment status of women was classified as employed or unemployed.

The information on selected household characteristics (i.e., walls, floor, water source, toilet facility, electricity source, and cooking stove) and assets (i.e., TV antenna, car, motorcycle, bicycle, horse, refrigerator, sewing machine, computer) was analyzed by principal component analysis (PCA) and summarized into a wealth index, which was weighted for the number of members residing in the household [30]. The scores were split into quintiles of wealth, where the first and fifth quintile represented the least poor and poorest, respectively.

### Analyses

Prevalence data were summarized as percent with 95% confidence intervals. The prevalence of poor self-rated health was analyzed by levels of food insecurity and other background characteristics. The lower-bound Cronbach’s alpha and its one-sided 95% confidence interval were computed to determine the internal consistency of the household food insecurity scale. A value of Cronbach’s alpha of 0.7 or higher was considered reliable [31]. Chi-square and Cochran-Armitage tests were used to assess associations and linear trends, respectively.

We considered women nested within communities. The self-rated health of women might be correlated within their communities. Therefore, we implemented a hierarchical modeling approach to analyze the association of food security as a capability with women’s self-rated health. The unadjusted and adjusted prevalence ratio and its 95% confidence interval were determined by random-intercept Poisson regression analyses [32].

Three models were developed. The first, the null model (no variables included in the model), identified variability across communities of the self-rated health outcome. In the second model, the exposure variable food insecurity was added, and finally, in the third model (full model), the covariates were incorporated to determine adjusted estimates and identify changes in variance between communities. The median prevalence ratio (MPR) was determined to assess the variability between communities by comparing two women from two randomly different communities [33]. The following formula was used for this purpose:

$$ MPR=\exp \left\{\sqrt{2\times \psi}\times {\upphi}^{-1}\left(\frac{3}{4}\right)\approx \exp \Big\{0.95\times \sqrt{\psi}\right\} $$

The value “exp” is equivalent to 2.7172, ψ is the contextual level variance, and ɸ^−1^(3/4) corresponds to the 75th centile of the cumulative distribution of the normal standard distribution with a mean 0 and a variance 1. If MPR is approximately equal to 1, then the community variation is not relevant. *P*-values < 0.05 were considered as significant for the association analyses. No evidence was found for multicollinearity based on the variance inflation factor (VIF) greater than 10 criteria (VIF = 1.61). The statistical analyzes were performed using Stata 15.0.

## Results

### Background characteristics

A total of 5866 completed records of non-pregnant women of reproductive age residing in 71 communities were included in the analyses after excluding missing records (4.7% in household records of food security and wealth). The average age was 29 (standard deviation 9.94) years and over half were 15–29 years. Few women were illiterate or had not completed primary education (5.5%) and few were employed (9.6%) (Table 1). The first component of the PCA explained 24.2% of the variability across the assets considered for the wealth index.

**Table 1:** Background characteristics of women of reproductive age, *Los Cuatro Santos*, Nicaragua, 2014

Characteristics	Level	n/N	(%)
Women’s age (years)			
	Less than 20	1231/5866	(20.9)
	20–29	1974/5866	(33.6)
	30–39	1504/5866	(25.6)
	40 or more	1157/5866	(19.7)
Women’s education			
	Completed Secondary or beyond	2740/5866	(46.7)
	Completed primary or not completed secondary	2798/5866	(47.7)
	Illiterate or not completed primary	328/5866	(5.5)
Women’s occupation			
	Employed	564/5866	(9.6)
	Unemployed	5302/5866	(90.3)
Wealth index			
	Fifth quintile (least poor)	1280/5866	(21.8)
	Fourth quintile	1237/5866	(21.0)
	Third quintile	1128/5866	(19.2)
	Second quintile	902/5866	(15.3)
	First quintile (poorest)	1319/5866	(22.4)
Food security			
	Food secure	634/5866	(10.8)
	Mildly food insecure	611/5866	(10.4)
	Moderately food insecure	2895/5866	(49.3)
	Severely food insecure	1726/5866	(29.4)

### Food insecurity

About half and one-third of household were moderately and severely food insecure respectively. Further, the proportion of women living with some level of food insecurity (mild, moderate, and severe) was 89.1% (95% CI: 86.1, 91.1). The Cronbach’s alpha of the household food insecurity scale was 0.90 with a 95% one-sided confidence interval of 0.90, indicating a high internal consistency.

### Self-rated health

**Fig. 1:**
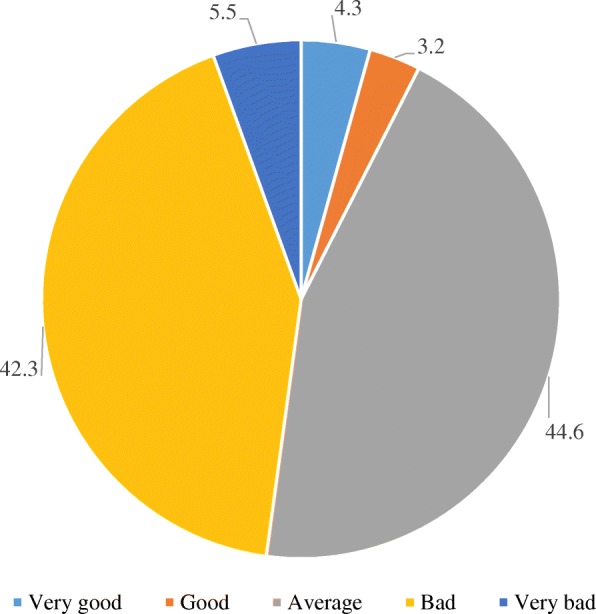
Self-related health percent distribution in women of reproductive age, *Los Cuatro Santos*, Nicaragua, 2014

**Fig. 2:**
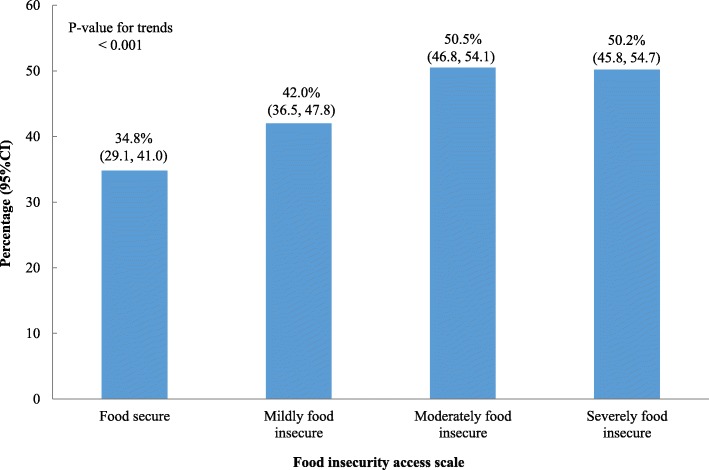
Poor self-related health distribution of women of reproductive age by food insecurity scale*, Los Cuatro Santos*, Nicaragua, 2014

Overall, 4.3% of women rated their health as very good, 3.2% good, 44.6% average, 42.3% bad and 5.5% very bad (Fig. 1). The proportion of poor SRH (bad + very bad) was 34.8% in women who were food secure, while among those living in moderate or severe food insecurity almost 50% had poor SRH (Fig. 2). The proportion of poor SRH was higher among older women, those with little education (illiterate or not completed primary school), unemployed, and the poorest (first wealth quintile) in comparison with the younger women, women with more education, employed, and least poor (fifth wealth quintile) (Table 2).

**Table 2:** Proportion poor self-rated health of women of reproductive age in different groups, *Los Cuatro Santos*, Nicaragua, 2014

Characteristics	Proportion of poor self-rated health (95% CI)	*p*-value; *p*-value for trends
Women’s age (years)		
Less than 20	33.8 (29.5, 38.4)	< 0.001; < 0.001
20–29	42.0 (38.2, 46.0)	
30–39	53.5 (49.9, 57.2)	
40 or more	65.1 (59.8, 70.1)	
Women’s education		
Completed Secondary or beyond	40.3 (36.4, 44.4)	< 0.001; < 0.001
Completed primary or not completed secondary	53.7 (50.4, 57.0)	
Illiterate or not completed primary	60.3 (55.3, 65.1)	
Women’s occupation		
Employed	40.7 (36.0, 45.6)	0.001
Unemployed	48.6 (45.2, 52.0)	
Wealth index		
Fifth quintile (least poor)	39.6 (34.8, 44.6)	
Fourth quintile	48.1 (43.7, 52.5)	
Third quintile	50.6 (46.0, 55.1)	
Second quintile	49.7 (45.2, 54.3)	
First quintile (poorest)	52.0 (47.4, 56.5)	< 0.001; < 0.001

### Association between food insecurity and self-rated health

The results of the regression analysis using community as a cluster variable are shown in Table 3. There was a relevant variance between communities. In the empty model, the median-prevalence ratio (MPR) displayed community factors associated with self-rated health (MPR = 1.51). In the food insecurity only model, the prevalence ratio of the association between food insecurity and poor self-rated health was 1.35 (95% CI: 1.18, 1.56). However, the MPR was 1.50, meaning that if two women with similar food insecurity were randomly chosen, the MPR between the woman residing in a community with higher propensity to poor SRH and the woman residing in the community with lower propensity to poor SRH was 1.50, suggesting some degree of heterogeneity between the communities. In the full model with food insecurity and covariates, the MPR decreased to 1.47 and the between-community variance of poor self-rated health remained significant. The associated prevalence ratio of food insecurity and poor self-rated health accounting for age, education, occupation, and wealth decreased to 1.27 (95% CI: 1.10, 1.48). Age and unemployment were significantly related to poor health. Increasing age and unemployment were positively associated with poor self-rated health.

**Table 3:** Factors associated with poor self-rated health of women of reproductive age. Random-coefficient Poisson regression, community considered as a cluster for analysis, *Los Cuatro Santos*, Nicaragua, 2014

Variables	Empty model	Intercept random model with food insecurity Prevalence ratio (95% confidence interval)	Intercept random model with food insecurity and covariates Prevalence ratio (95% confidence interval)
Food security			
Food secure		1	1
Food insecure		1.35 (1.18, 1.56)	1.27 (1.10, 1.48)
Women’s age (years)			
Less than 20			1
20–29			1.24 (1.10, 1.40)
30–39			1.55 (1.37, 1.76)
40 or more			1.93 (1.69, 2.20)
Women’s education			
Completed Secondary or beyond			1
Completed primary or not completed secondary			1.06 (0.90, 1.25)
Illiterate or not completed primary			1.08 (0.99, 1.18)
Women’s occupation			
Employed			1
Unemployed			1.20 (1.04, 1.39)
Wealth index			
Fifth quintile (least poor)			1
Fourth quintile			1.11 (0.97,1.28)
Third quintile			1.11 (0.96, 1.28)
Second quintile			1.13 (0.99, 1.29)
First quintile (poorest)			1.13 (1.00, 1.28)
Measure of clustering			
Contextual level standard deviation (standard error)	0.1918 (0.02828)	0.1839 (0.02828)	0.1702 (0.02841)
Median prevalence ratio	1.51	1.50	1.47
Likelihood ratio test (*p*-value)	48.69 (< 0.001)	40.91 (< 0.001)	30.02 (< 0.001)

## Discussion

Food insecurity affected nine out of ten households in *Los Cuatro Santos*. The results also suggested lower self-rated health at higher levels of food insecurity. This association was found across the different communities.

In the area, food insecurity was still at a high level, although reduced over the last five years. For instance, the proportion of severely food insecure households dropped from 36% in 2009 to 29% in 2014 [27]. Our group reported a poverty reduction over the period of 2003–2009, which may be linked to the improvement in food security, as the food insecurity scale shows results related to economic and physical access to food [34].

Women who were employed and living in wealthier households had better self-rated health. Self-rated health was reduced by age, which has been found in many settings [35]. Despite a high level of secondary education, unemployment was still high. The lack of job opportunities might limit the ability of women to gain autonomy and manage their own resources for health and well-being. Therefore, the relationship between unemployment, low socioeconomic status and food insecurity may reflect the reduced capacity to buy and get access to food. Self-rated health was lower by decreasing wealth as shown in other contexts [17, 36]. The association between food insecurity and self-rated health might even better reflect this relationship. This result is consistent with other studies performed in similar settings [36, 37]. In poor settings, a larger share of the household budget is allocated to food or health care. However, women in rural low-income settings might have less control of economic resources than men [38]. This inequality might limit their ability to make important decisions regarding their well-being and use of health services.

In this area where access to food is insecure, this must be seen as a lack of capabilities. As shown by Sen and others, when this happens the dignity of poor people is damaged [39]. In our study sample, at least four-fifth of women experienced anxiety for food supply in the household. Anxiety is a physiological distress potentially related to poor quality sleep and depression. For a mother, this situation might affect her capability to get sufficient food for herself and her dependent children. Along this line, food insecurity is also reported to be shameful [13, 28]. With this in mind, we suggest that food security is an important facet of poverty according to the capability theory. It measures an important missing capability directly related to health by missing nutrients, but also indirectly enhancing the potential to be exposed to risks of non-communicable diseases (e.g., unhealthy diet, obesity) [40].

In our study, at least 80% of women perceived lack of access to diverse foods for consumption (data not shown, question 3 HFIAS), a proxy assessment of food quality. Having the resources and opportunities to consume a diverse diet increases the prospects for good nutritional and health outcomes. Nevertheless, it is acknowledged that healthy foods as part of a diverse diet are more expensive than unhealthy foods [41]. This reflects another missing capability when people are unable to meet important nutritional recommendations. We observed at least one-third of women with poor self-rated health in the food secure group. This finding might indicate that capabilities such as maintaining diet quality are not met, but it requires more research.

The data in *Los Cuatro Santos* HDSS is reportedly of good quality [34]. In the analyses, potential cluster effects were accounted for. The food insecurity scale showed a high reliability. Non-participation rate was very low, eliminating any potential selection bias. The cross-sectional design, however, prevents causal inferences. We did not include data on women’s diagnoses or health problems, which could have shed light on the specific conditions behind the general statements on self-rated health. The findings might be representative of western rural Nicaragua. Most likely other poor communities in the Latin American and Caribbean countries share similar characteristics and associations between food insecurity and perceived well-being.

Local individual-level data have advantages in comparison with the ecological national data frequently used by international agencies. Although the study of causal pathways between different capabilities and health calls for longitudinal analyses, the local level individual data do not suffer from the obvious risks of spurious associations when analyzing ecological data from national statistics. Local data may also be used to initiate and reinforce grassroots strategies to promote development and combat health inequalities, which frequently has been the case in the study area [24, 42].

## Conclusions

A high prevalence of food insecurity was associated with poor self-rated health among women in rural northern Nicaragua. Although the mechanisms remain unclear, we analyzed the limited access to food as an important dimension of poverty using the capability approach. Future research is needed to link social development, health, and well-being using longitudinal designs and including other capability indicators.

## Abbreviations

CI: Confidence Interval
HDSS: Health and Demographic Surveillance System
HFIAS: Household Food Insecurity Access Scale
LMIC: Low- and Middle-Income Countries
MPR: Median Prevalence Ratio
PCA: Principal Component Analysis
PR: Prevalence Ratio
SDG: Sustainable Development Goals
SRH: Self-Rated Health
VIF: Variance Inflation Factor
